# Anataselike Grain Boundary Structure in Rutile Titanium Dioxide

**DOI:** 10.1021/acs.nanolett.0c04564

**Published:** 2021-03-31

**Authors:** Georg Schusteritsch, Ryo Ishikawa, Abdul Razak Elmaslmane, Kazutoshi Inoue, Keith P. McKenna, Yuichi Ikuhara, Chris J. Pickard

**Affiliations:** † Department of Materials Science and Metallurgy, University of Cambridge, 27 Charles Babbage Road, Cambridge CB3 0FS, United Kingdom; ‡ Advanced Institute for Materials Research, Tohoku University, 2-1-1 Katahira, Aoba, Sendai 980-8577, Japan; ¶ Institute of Engineering Innovation, The University of Tokyo, 2-11-16 Tokyo 113-8656, Japan; § Japan Science and Technology Agency, PRESTO, Kawaguchi, Saitama 332-0012, Japan; ∥ Department of Physics, University of York, Heslington, York YO10 5DD, United Kingdom; ⊥ Nanostructures Research Laboratory, Japan Fine Ceramics Center, 2-4-1 Nagoya 456-8587, Japan

**Keywords:** grain boundary structure, STEM, structure prediction, DFT, TiO_2_

## Abstract

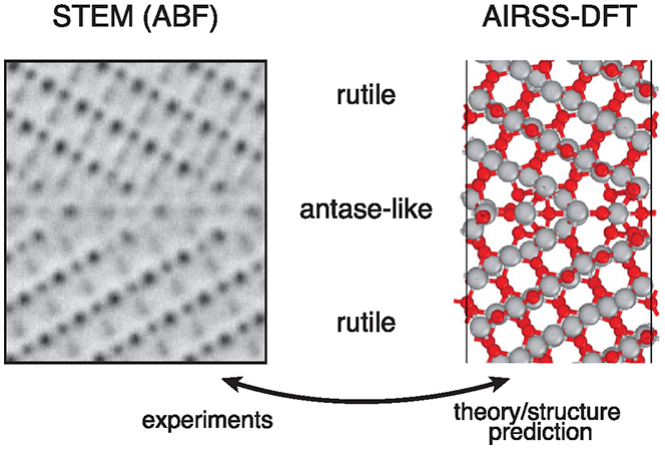

The formation of nanoscale phases at grain boundaries in polycrystalline materials has attracted much attention, since it offers a route toward targeted and controlled design of interface properties. However, understanding structure–property relationships at these complex interfacial defects is hampered by the great challenge of accurately determining their atomic structure. Here, we combine advanced electron microscopy together with *ab initio* random structure searching to determine the atomic structure of an experimentally fabricated Σ13 (221) [11̅0] grain boundary in rutile TiO_2_. Through careful analysis of the atomic structure and complementary electron energy-loss spectroscopy analysis we identify the existence of a unique nanoscale phase at the grain boundary with striking similarities to the bulk anatase crystal structure. Our results show a path to embed nanoscale anatase into rutile TiO_2_ and showcase how the atomic structure of even complex internal interfaces can be accurately determined using a combined theoretical and experimental approach.

## Introduction

Virtually all naturally occurring and most artificially fabricated materials are polycrystalline, with interfaces forming between the different grains, so-called grain boundaries. These grain boundaries and the processes occurring at them often strongly influence the mechanical, electronic, optical, and thermal transport properties of the macroscopic system^1^ and thus play a crucial role in the discovery of novel physics and the design of next-generation devices. One area of particular interest is understanding the formation and subsequent possible transformations of unusual phases forming at grain boundaries.^2−5^ This has received much attention recently, and a better understanding of grain boundary phases, that is the local atomic structure at grain boundaries, has been described as one of the eight grand challenges in ceramics science.^3^ Ceramics are prevalent in many devices but linking material performance with the atomic structure of the interfaces has remained a great challenge. We note some authors have proposed the term complexion for certain types of grain boundary phases, but here we prefer not to use this term to avoid introduction of unnecessary terminology and ambiguities in its definition when the interfacial phase is closely related to a stable bulk phase.^6,7^ Our understanding of and ability to predict the properties of interfaces and thus the design of materials via engineering the interfaces within them is still mostly lacking, largely hampered by our inability to predict the atomic structure of interfaces and unravel the structure–property relation at grain boundaries for anything but the easiest systems.

Within the large set of ceramic compounds a system of particular interest is TiO_2_: It finds application in for instance photocatalysis, catalysis, solar cells, and gas sensors.^8−10^ At ambient conditions it can be found to naturally occur in the rutile, anatase, and brookite phases.^11^ Other higher pressure phases have been successfully synthesized, in particular, the columbite, fluorite, pyrite, baddeleyite, and cotunnite structures,^11^ where the atomic structure can be used to control the properties for device applications.^12^ Based on experiments it is generally believed that rutile is the thermodynamically most stable phase at ambient conditions, though recent theoretical work employing high accuracy quantum Monte Carlo calculations indicates a different ordering that puts anatase marginally as the most stable of the two polymorphs.^13^ Despite this discrepancy, the atomic structures of crystalline TiO_2_ and their properties are largely established. Much less is known about the grain boundary phases that can form within TiO_2_, but the rich polymorphism of crystalline TiO_2_ with several energetically accessible structures points to an equally rich phase space for grain boundary phases. Some work on the properties of specific TiO_2_ grain boundaries does exist already, indicating that the exact structure the grain boundary phases possess strongly affects the bulk behavior.^2,14−19^ Sun et al. for instance previously studied the Σ3 (112) [11̅0] grain boundary in TiO_2_ and showed grain boundary phase transformations induced by heat and atmospheric treatment.^2^ Gao et al. studied the Σ5 (210) [001] grain boundary in TiO_2_ and found a narrowing of the band gap for this particular grain boundary for nonstoichiometric conditions.^19^ Twin boundaries in nanocrystalline anatase TiO_2_ have also been suggested to enable nucleation of rutile TiO_2_ in the anatase to rutile phase transformation.^20,21^ Designing specific properties by altering the atomic structure or engineering specific grain boundary phases is still hampered by the need for more advanced methods to determine the atomic structure and thereby unravel the structure–property relationship. Transmission electron microscopy, though in principle able to probe the atomic structure and identify the different atomic species at interfaces, faces the challenge of determining the atomic structure of a grain boundary in three dimensions based on two-dimensional images. At the same time conventional computational approaches are often unable to determine the atomic structures of any but the simplest interfaces in oxides. Designing materials with grain boundaries of a specific phase remains a significant obstacle and new approaches need to be established to advance this field toward “interfaces by design”.

We address this major challenge in ceramic science using a combined theory and experimental approach combining atomic-resolution scanning transmission electron microscopy (STEM) with state of the art computational interface structure prediction techniques. Aberration-corrected high-angle annular dark-field (HAADF)^22^ and annular bright-field (ABF)^23,24^ STEM are used to obtain atomic-resolution images of a Σ13 grain boundary in rutile TiO_2_, providing a starting point to define constraints to perform structure prediction calculations, using high-throughput density functional theory (DFT) calculations within the *ab initio* random structure searching (AIRSS) framework.^25−27^ These calculations are in turn fed back to experiments via image simulations and refined to match the experimental results, illustrating the important synergy between experiments and theory to accurately determine grain boundary phases in such complex systems as in the Σ13 (221) [11̅0] GB in TiO_2_ we study here. Bond-orientational order parameter analysis based on the proposed atomic structure as well as experimental and theoretical electron energy-loss spectroscopy (EELS) show that the grain boundary phase exhibits anatase character embedded in bulk rutile TiO_2_. The results of this work are thus twofold: We show a state of the art combined theoretical and experimental approach toward unravelling the structure–property challenge of grain boundaries in ceramics. At the same time it demonstrates that it is possible to fabricate well-defined anatase(like) regions within rutile TiO_2_, opening the way to combine these two phases in a well-defined controllable manner. The latter could have significant implications for the realization of next-generation devices requiring the presence of both anatase and rutile TiO_2_ in a very controlled structure, while the former demonstrates how other unique and complicated grain boundary phases can be explored efficiently to high accuracy.

## Results and Discussion

### Microstructure of the TiO_2_ Bicrystal

Generally, polycrystalline materials consist of grain boundaries with many different orientations, including both high symmetry and random grain boundaries. We focus here on identifying to high accuracy the grain boundary phases of a high symmetry grain boundary in the form of a bicrystal. In particular, we fabricate a Σ13 (221) [11̅0] grain boundary with a tilt angle of 57.5° in rutile TiO_2_ using the bicrystal fabrication technique by combining two single crystals of rutile TiO_2_ by high temperature solid-state diffusion bonding. The crystallographic orientation of the bicrystal was first evaluated by selected-area electron diffraction analysis, and we confirmed that the present bicrystal has a Σ13 (221) [11̅0] orientation relationship (see Supplementary Figure 5). Low-magnification ABF-STEM images show perfect joining of the bicrystal over a long range (Supplementary Figure 1). No significant dopants were recognized in this grain boundary via EELS and Z-contrast STEM. Focusing on a high symmetry grain boundary makes imaging and identifying the atomic coordinates at and near the grain boundary more feasible and thus enables comparison of the theoretical and experimental results for the grain boundary phase at the interface.

### Atomic-Scale Imaging of the Grain Boundary

**Figure 1:**
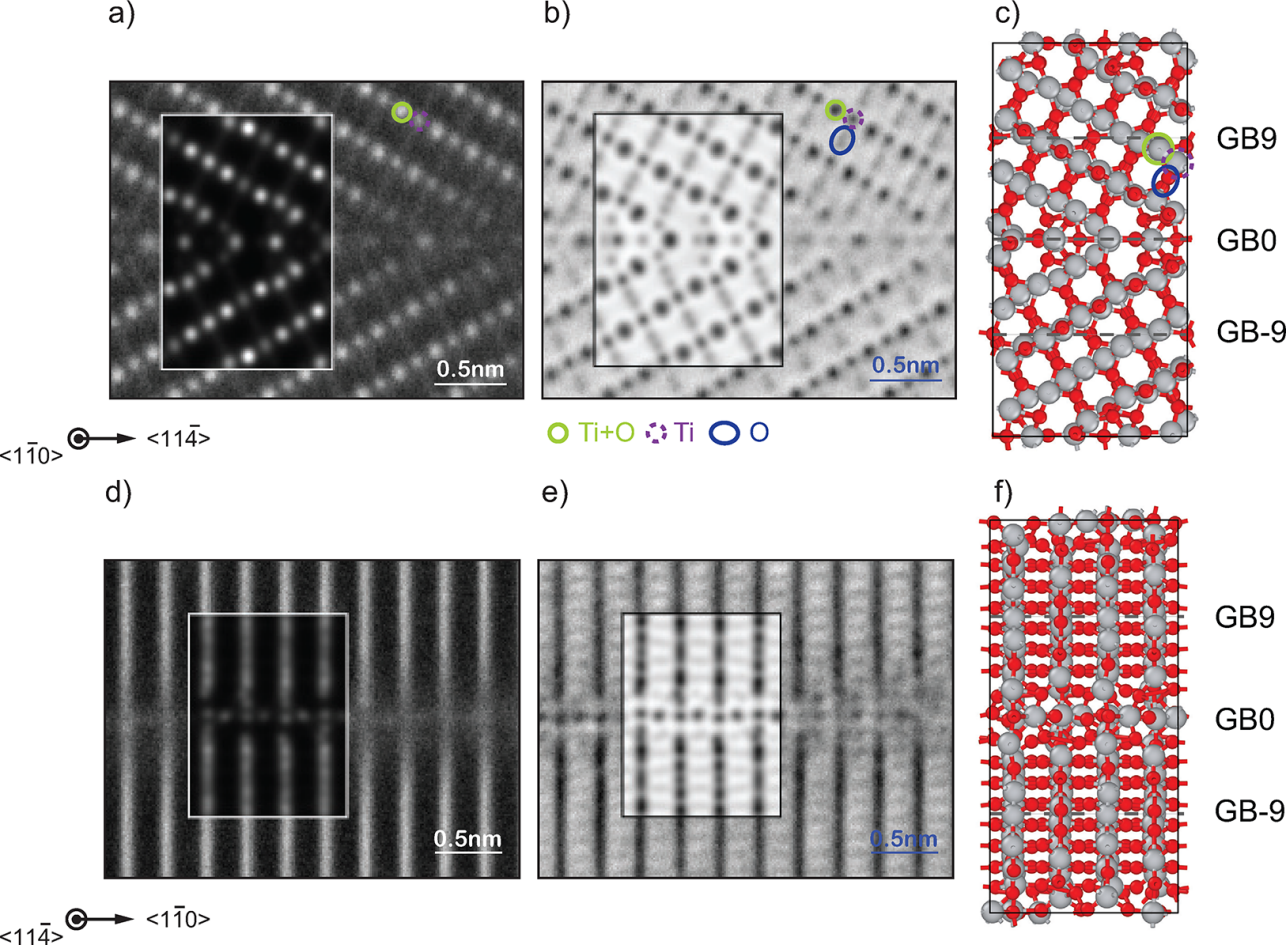
(a) Atomic-resolution HAADF. (b) ABF STEM images and (c) the theoretical atomic structure (A*) of the Σ13 grain boundary along the [11̅0] direction. (d) Atom-resolved HAADF. (e) ABF STEM images and (f) the theoretical atomic structure (A*) of the Σ13 grain boundary along the [114̅] direction. Image simulations based on the atomic structure of structure A* are shown as insets in the experimental HAADF and ABF STEM images. Gray and red spheres represent Ti and O atoms, respectively. Columns of Ti + O, Ti, and O atoms are marked by solid green circles, dashed purple circles, and blue ellipsoids, respectively. Images of the theoretical atomic structures are based on images generated using OVITO.^28^

To ascertain the atomic structure of the grain boundary, we initially consider HAADF STEM images along the ⟨11̅0⟩ and ⟨114̅⟩ directions, shown in Figure 1a and d, respectively (large-scale images without insets of the image simulation in Supplementary Figure 2). The image intensity in HAADF STEM mode of an atomic column is approximately proportional to the atomic number to the power of 1.7, *Z*^1.7^, that is brighter spots in the images are indicative of heavier atoms in the region or similarly a greater atomic density. In the bulk region of the bicrystal for images along ⟨11̅0⟩ (Figure 1a) this lets us identify Ti–O columns as the brighter spots in comparison to the Ti-only atomic columns which are less bright (green circles and purple dashed circles, respectively). For the HAADF STEM images along ⟨114̅⟩, we see continuous lines of high intensity perpendicular to the grain boundary interface; these are Ti and O atoms overlapping as expected for rutile TiO_2_ viewed along this direction, making identifying individual columns of atoms impossible. We further elucidate the atomic structure of our bicrystal by applying the ABF STEM imaging technique. This allows for simultaneous imaging of both light and heavy atomic species. In the case of ABF STEM images, dark spots are indicative of the presence of atoms and the Ti + O and Ti columns seen in the HAADF STEM images can be clearly identified again (Figure 1b and e). We can further identify columns of O atoms between the rows of Ti + O and Ti atoms for ⟨11̅0⟩ and between rows of Ti + O for ⟨114̅⟩. Although identification of the atomic positions for rutile TiO_2_ in the region away from the interface is relatively straightforward given our HAADF and ABF STEM images, identifying the atomic positions and respective species at the interface with high certainty is challenging.

### Identification of Grain Boundary Region via Computational Structure Prediction

To identify the unresolved atomic structure of the grain boundary, we perform AIRSS calculations.^25,26^ Using this approach to identify the low energy atomic configurations at interfaces has previously been successfully applied to grain boundaries in both graphene and SrTiO_3_^27^ as well as heterostructure interfaces.^29^ The details of the approach can be found in ref (27), with further computational details for this system in the Supporting Information.

**Figure 2:**
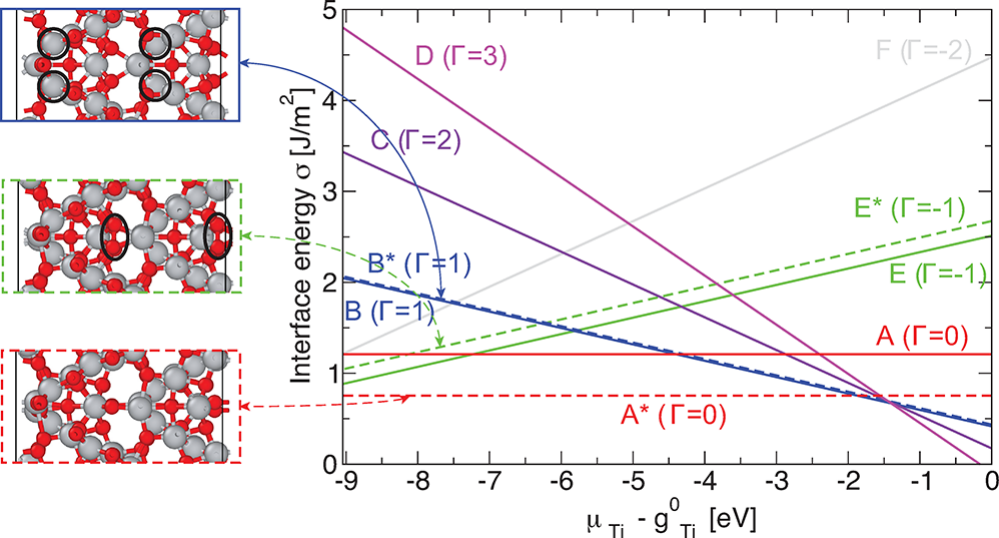
Interfacial energy for the different theoretical structures. The lowest energy stoichiometric structure A* (shown as a dashed red line) is found to match the experimental images. The atomic structures of configurations B*, E*, and A* are shown. The Ti atoms one layer away from the center of the grain boundary (GB ± 1) are marked by black circles for structure B*. Similarly, black ellipses mark the O–O pairs at the grain boundary of structure E*. All dashed lines shown and equally the structure names with an asterisk are for structures with twice the lattice vector along [114̅]. Gray and red spheres represent Ti and O atoms, respectively. Images of the theoretical atomic structures are based on images generated using OVITO.^28^

We consider the STEM images to constrain the search (details in the Supporting Information) and determine the excess interfacial energy, σ, as a function of the Ti chemical potential to find the low energy structures as shown in Figure 2. Using structure searching we initially find structures A–E (solid lines in Figure 2). By comparing to the experimental results we are able to further alter the low-energy structures (details in the Supporting Information), resulting in structure A*, a stoichiometric structure, significantly lower in energy over a large range of chemical potential than all other structures. Image simulations and direct comparison shows this structure matches the experimental images exceedingly well (Figure 1). Careful inspection between the HAADF as well as the ABF images and the theoretically determined structures, including image simulations shown in Figure 1, together with our interface energy calculations leads us to propose structures A* as the likeliest formed in the bicrystal.

Viewed along the [11̅0] direction we first notice that at the center of the grain boundary the columns of Ti, Ti + O and O atoms correspond to a very high degree with the signal from the experimental images. At the same time we see that the rows of Ti + O and Ti columns line up very accurately, reaching far into the bulk, further indicating that our predicted structure at the center of the grain boundary must closely match the experimentally fabricated sample. We also see good correspondence along the [114̅] direction: In that case we notice that the rows of Ti + O atoms perpendicular to the (221) plane are connected at the grain boundary plane with an extra set of atoms between the rows of Ti + O atoms. This is a peculiar configuration, very different to the previously found structure of the Σ3 (112) [11̅0] grain boundary in TiO_2_ where rows of Ti + O atoms are connected in a continuous fashion, not as significantly interrupting the bulk rutile phase.^2^ We find that these extra Ti atoms play a crucial role in giving this grain boundary a very different character at the center of the grain boundary.

### Experimental and Theoretical EELS

**Figure 3:**
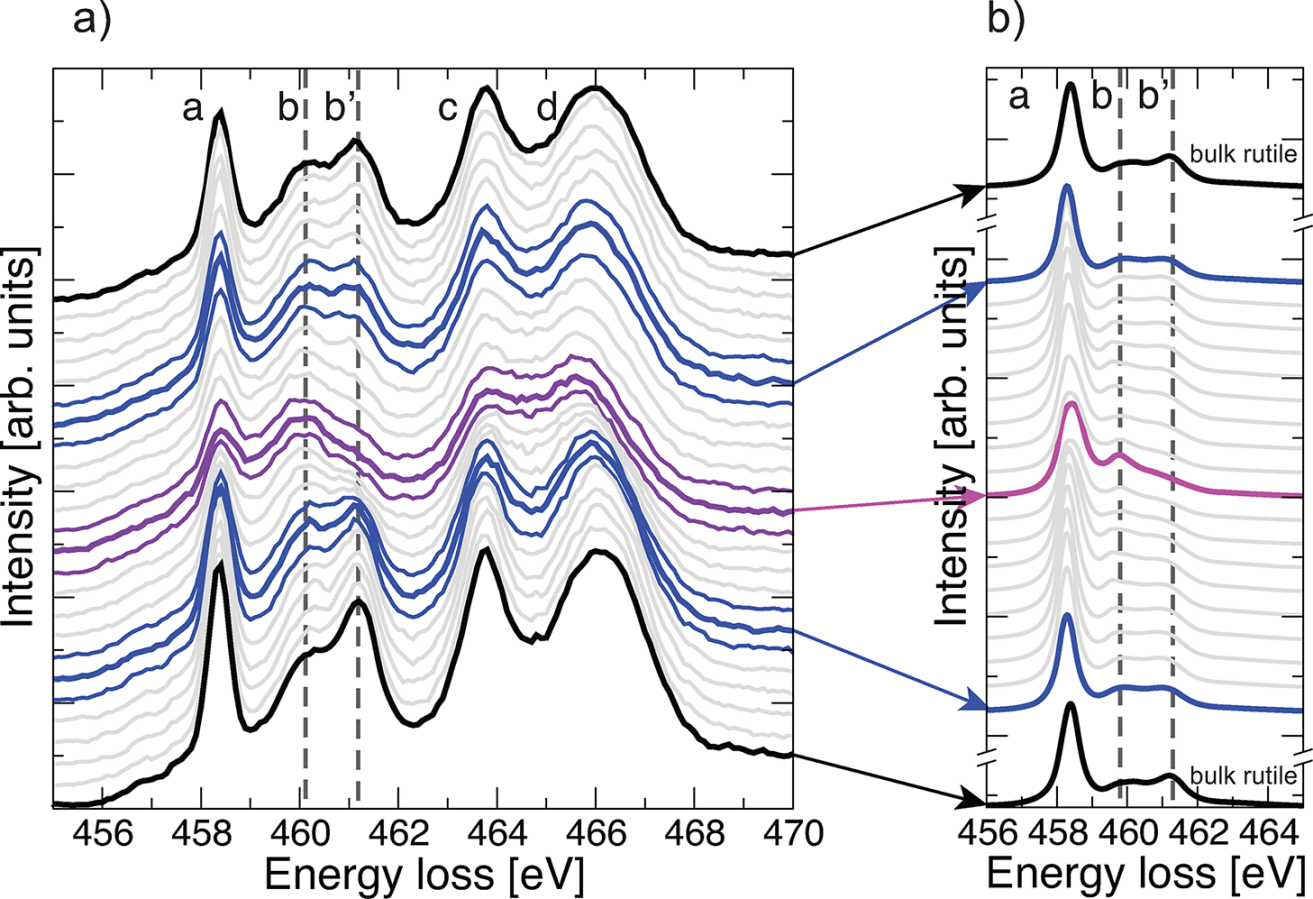
(a) Experimental EELS profiles of the Ti *L*_2,3_ edge of the Σ13 grain boundary recorded parallel to the grain boundary plane. The signal intensity for peak b increases as the grain boundary is approached, while that of b’ decreases—this is indicative of a change from a rutilelike to anataselike signal as the grain boundary is approached. (b) Calculated EELS of the determined Σ13 structure. A changeover of the peak intensities corresponding to a transition from rutile to anatase character is detected as the grain boundary is approached, consistent with the trend seen in experiments. Computed energies are shifted based on the position of experimental peak a.

To investigate the apparent change from bulk rutile to a confined region of anatase character at the grain boundary and its effect on the electronic structure, we perform EELS measurements and compare them to the calculated signal based on our proposed atomic structure. We focus here on the *L*_2,3_ Ti edge since bulk rutile and anatase show two very distinct different signals in the *L*_2,3_ edge where, of the five major peaks, peaks b and b’ switch in magnitude (Figure 3 and Supplementary Figure 9) allowing for identification between the two bulk phases.^30,31^

Figure 3a shows a line scan perpendicular to the grain boundary. In the regions ≳1 nm away from the grain boundary the *L*_2,3_ edge clearly shows bulk rutile character with peak b significantly lower in magnitude than peak b’. As the grain boundary is approached the signal gradually changes with the relative magnitude of peaks b and b’ switching such that the signal at the grain boundary clearly exhibits anatase character. We also calculate the EELS spectrum of our predicted structure A*, using nonrelativistic, one-electron calculations which ignore many-electron and multiplet effects as well as spin–orbit coupling. The overall trends for peaks a, b, and b’ within this approximation are preserved, allowing us to distinguish bulk rutile and anatase phases (see the Supporting Information). We thus calculate the *L*_2,3_ edge for each layer and assuming Gaussian broadening with fwhm of 2 Å to approximate the beam in the experimental EELS measurement. This is shown in Figure 3b and the same behavior as for the experimental results can be observed, that is a change from approximately rutile to anatase character. The signal at GB = ±9 is not exactly the same as the bulk signal of rutile TiO_2_, but this can easily be explained by the fact that we use a setup in our calculations with two periodic grain boundaries, i.e. it is likely there was not enough space to fully relax the structure to its bulk structure, since we did not constrain the atoms to their ideal bulk rutile positions between the two grain boundaries.

### Bond-Orientational Order Parameters

**Figure 4:**
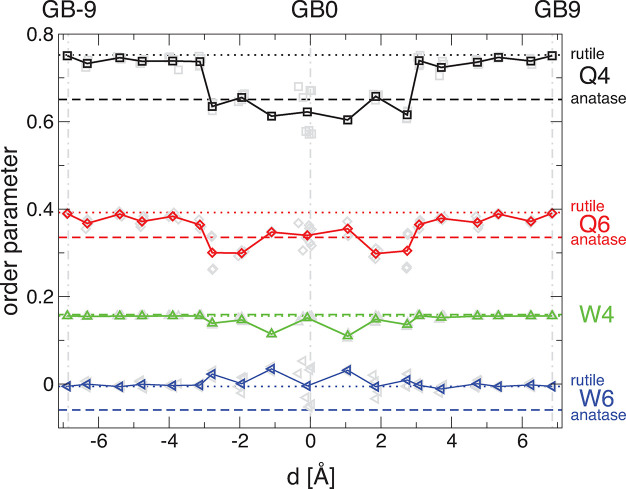
Bond-orientiational order parameter for all Ti atoms of the GB structure showing the bond character to the nearest neighbor O atoms. Dotted black, red, green, and blue lines show the values of the bond-orientational order parameters Q4, Q6, W4, and W6, respectively, for bulk rutile TiO_2_. Dashed lines with the same color coding convention show the same for bulk anatase TiO_2_. Black squares, red diamonds, green upward triangles, and blue leftward triangles show the average Q4, Q6, W4, and W6 bond-orientational order parameters, respectively, for each layer of the grain boundary, with the individual contributions shown as light gray symbols for each order parameter in the background (solid lines as guide for the eye only). The center of the grain boundary is marked as GB0 with a dashed–dotted gray line at *d* = 0. The layer furthest from the center of the grain boundary is marked as GB9 similarly with a dashed–dotted gray line.

To further investigate the structural characteristics of the grain boundary phase we first consider the bond-orientational order parameters as introduced by Steinhardt et al.^32,33^Figure 4 shows a trend from rutile character (dotted lines) at around GB ± 9 to more anatase character (dashed lines) starting at around GB ± 3 for the Q4 and Q6 parameters. The difference between anatase and rutile for W4 is too small to draw any conclusion, while the signal for W6 remains more rutilelike on average, with some outliers at the very center of the grain boundary—shown as light blue triangles; these are the special Ti atoms between the rows of atoms.

Clearly, our results for the Steinhardt bond-orientational order parameter of the predicted atomic structure are consistent with our findings based on the experimental and theoretical EELS profile as a function of distance from the grain boundary. The latter are both experimentally cost-intensive and computationally expensive, respectively, while analysis of the Steinhardt bond-orientational order parameters is relatively straightforward in comparison. This analysis thus offers a convenient approach for high-throughput analysis of the grain boundary phases of theoretical grain boundary structures.

**Figure 5:**
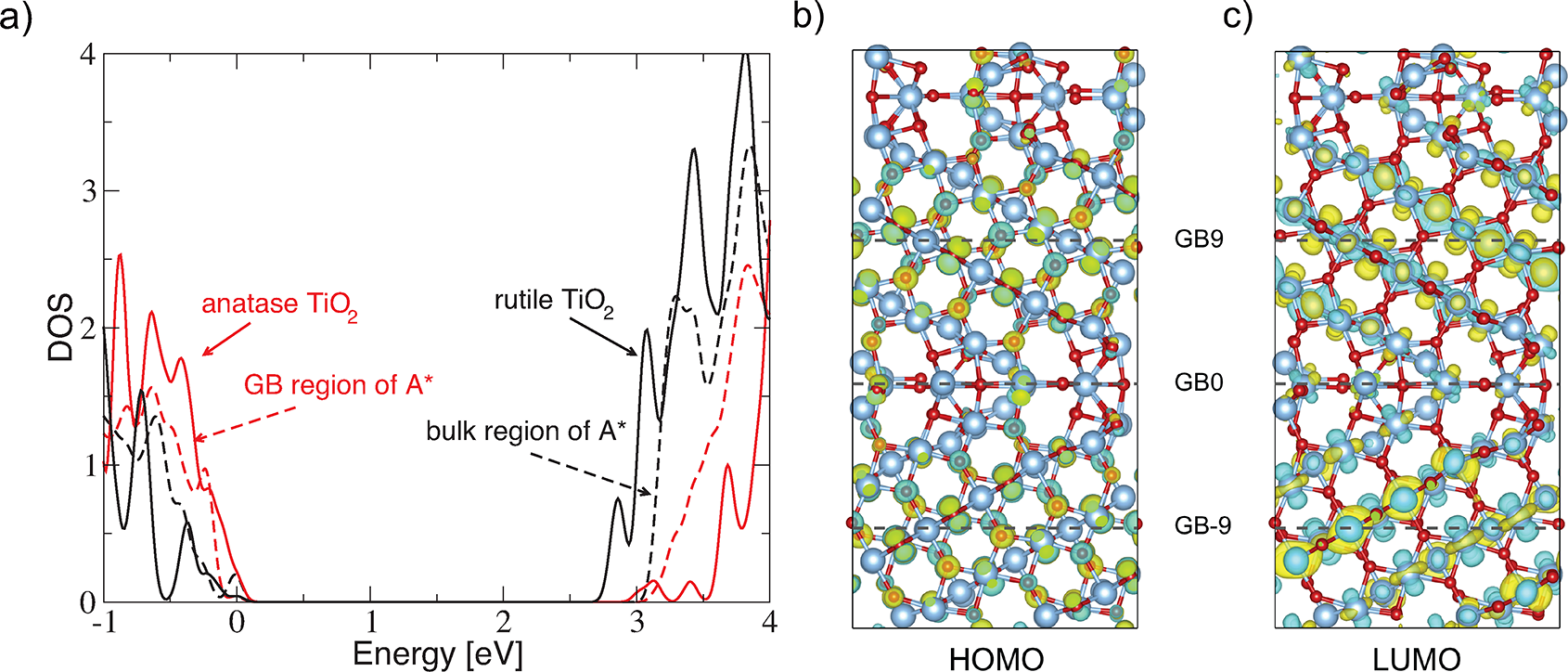
(a) Total and projected density of states for crystalline anatase and rutile and of the grain boundary structure A*. Shown also are the isosurfaces of the highest occupied (b) and lowest unoccupied molecular orbitals (c).

We conclude our discussion by considering the electronic structure of the grain boundary and the region surrounding it (Figure 5). We employ the method outlined in ref (34) (details in the Supporting Information). By considering the projected density of states of atoms within a narrow region of the grain boundary of structure A* (all atoms within GB ± 2 red dashed line), we find that its character closely follows that of crystalline anatase TiO_2_. When considering the density of states in the more bulklike region (GB ± 7, GB ± 8, and GB ± 9 black dashed line) we find that the behavior becomes less anataselike. Full rutile TiO_2_ behavior is not recovered, but this is likely an artifact of the inherently limited size of the supercell employed here. The overall behavior thus closely follows that seen in our EELS calculations and measurements and again shows the anatase character of this TiO_2_ grain boundary.

We further find that the top of the valence band and bottom of the conduction band are both dominated by bulk states (Figure 5a). This is also reflected in the HOMO and LUMO states which show electrons and holes confined primarily to the bulk of the grains (Figure 5b and c). This suggests that photo-excited charge carriers face a barrier to cross this type of grain boundary, which would limit mobility, consistent with the fact that the mobility of polycrystalline TiO_2_ is known to be much lower than that of single crystals.

## Conclusion

In this work we demonstrate how an approach combining state of the art computational methods and experimental imaging spectroscopy techniques can be used to address the challenge of determining the atomic structure of interfaces—a great obstacle that has for long hampered understanding of the structure–property relationship of interfaces and is key in advancing materials by design beyond purely crystalline systems toward complicated systems of importance to many devices. HAADF and ABF STEM images allow us to constrain the interface search we perform using AIRSS, thereby reducing the search phase space. This enables us to efficiently find the atomic structure of the Σ13 (221) [11̅0] GB in TiO_2_, allowing us to study its unusual grain boundary phase. We find both experimental evidence from EELS as well as evidence from EELS calculations based on our proposed structure that the fabricated structure shows a confined region that exhibits anatase character at the grain boundary embedded in rutile TiO_2_. This is further confirmed by analysis of the bond-orientational order parameters of our theoretical structures—the latter illustrates a path toward efficient prescreening the grain boundary phases of other interfaces. Finally, we directly investigate the band gap and electronic structure of the grain boundary and again find that the grain boundary exhibits anatase character. Beyond the importance to the fundamental understanding of unusual grain boundary phases, our work shows that it is possible to embed regions of anatase character into rutile TiO_2_ via grain boundary fabrication; this has clear technological implications. In general, we show that our combined computational and experimental approach allows for successful determination of complicated grain boundary phases that are otherwise not accessible separately: This emphasizes that the whole of combining cutting edge experimental with cutting edge structure prediction techniques is greater than the sum of its parts, thereby making otherwise currently inaccessible systems possible to resolve for future interface engineering.

## Supporting Information Available

Further details of the experimental and computational method (PDF)
All crystal structures in CIF format (ZIP)
