# Supplementary material for: Anataselike Grain Boundary Structure in Rutile Titanium Dioxide
Source: Nano Lett. 2021 Mar 31;21(7):2745–51. doi: 10.1021/acs.nanolett.0c04564 (PMC8155194; doi:10.1021/acs.nanolett.0c04564)
Supplement: Supplementary file 1 — nl0c04564_si_001.pdf [file nl0c04564_si_001.pdf]

Anatase-like grain boundary structure in rutile titanium dioxide

Georg Schusteritsch,[*,†,‡] Ryo Ishikawa,[¶,§] Abdul Razak Elmaslmane,[‖] Kazutoshi Inoue,[§,‡] Keith P. McKenna,[‖] Yuichi Ikuhara,[*,¶,⊥,‡] and Chris J. Pickard[*,†,‡]

† *Department of Materials Science and Metallurgy, University of Cambridge, 27 Charles Babbage Road, Cambridge CB3 0FS, United Kingdom*

‡ *Advanced Institute for Materials Research, Tohoku University 2-1-1 Katahira, Aoba, Sendai, 980-8577, Japan*

¶ *Institute of Engineering Innovation, The University of Tokyo, 2-11-16 Tokyo 113-8656, Japan*

§ *Japan Science and Technology Agency, PRESTO, Kawaguchi, Saitama 332-0012, Japan*

‖ *Department of Physics, University of York, Heslington, York YO10 5DD, United Kingdom*

⊥ *Nanostructures Research Laboratory, Japan Fine Ceramics Center, 2-4-1 Nagoya 456-8587, Japan*

Supporting Information

Fabrication of bicrystal

The $\Sigma 13\,(221)\,[1\bar{1}0]$ grain boundaries of TiO_2 were fabricated by thermal diffusion bonding technique. Two pristine TiO_2 single crystal blocks of 99.9% purity (Shinkosha, Ltd. Tokyo, Japan) were first cut exactly along the (221) plane of the TiO_2 crystal. Then, two sin-

gle crystals were precisely joined face-to-face to fulfill the bicrystallographic relationship of $(221)\,[1\bar{1}0]_{\text{upper}} \parallel (221)\,[1\bar{1}0]_{\text{lower}}$, which were heated at 1773 K for 10 h in air.

Scanning Transmission Electron Microscopy

The TiO_2 bicrystal was cut and mechanically polished to be less than 100 µm. Then the sample was thinned via conventional Ar ion-beam thinning method to obtain electron transparency. To significantly suppress the surface damage by Ar ion-beam, we finally used 0.5 kV accelerate voltage for Ar ion-thinning. The atomic-resolution ABF and HAADF STEM images were taken by ARM300CF (JEOL) installed at the University of Tokyo, operating at 300 kV and equipped with an ETA aberration corrector and a cold field emission gun. The illumination semi-angle is 24 mrad and the detector collection semi-angles are $12 - 24$ mrad and $64 - 200$ mrad for ABF and HAADF-STEM, respectively.

EEL spectra were obtained by using a Wien filter monochromated aberration corrected STEM (JEM-2400FCS, JEOL) installed at Japan Fine Ceramics Center, operating at 60 kV and equipped with an EEL spectrometer of GIF Tridiem ERS (Gatan). The dispersion energy per channel is 0.1 eV. The background signals of Ti-$L_{2,3}$ edge spectra were subtracted by power law fitting.

The EEL spectra in Fig.3 a) of the main text have been performed over a range of 25 Å perpendicularly to the grain boundary interface, with a distance of 1 Å between them.

Additional low-magnification HAADF-STEM image showing perfect joining of the bicrystal over a long range is shown in Figure 1.

Figure 2 and 3 show the atomic-scale HAADF and ABF STEM images, respectively, that the cropped images of the main manuscript are based on.

Image Simulations

The image simulations were performed using a multislice algorithm with the frozen phonon model (TEMSIM package[1]) for a 300 kV probe, where the illumination angle and detector

collection angles are the same as the experiment. To compensate the spatial incoherence of the microscope, the simulated images were convolved with a Gaussian finite source size.

Computational method

We apply *ab initio* random structure searching (AIRSS)[2,3] to the structure prediction of interfaces as previously introduced in ref. 4. The method relies on high-throughput density functional theory (DFT) calculations performed using CASTEP[5] and allows for automatic searching of the atomic structure of the interface with variable stoichiometry. During searching coarse parameters are chosen for the DFT calculations, which are then refined for our final results. For searching a plane wave cutoff of 350 eV was chosen, we use a Monkhorst-Pack grid of size $2 \times 2 \times 1$ and ultra-soft pseudo-potentials that treat the valance electrons for the $3d^2$, $4s^2$ states for Ti and the $2s^2$ $2p^4$ states for O. The XC energy is approximated by the PBE generalized gradient approximation (GGA).[6] All ionic positions are relaxed such that all forces are less than 0.05 eV/Å in magnitude using the limited-memory Broyden-Fletcher-Goldfarb-Shanno (L-BFGS) method. Our final calculations are based on much stricter parameters, namely a plane wave cutoff of 625 eV, a Monkhorst-Pack grid of $2 \times 3 \times 1$ (equivalent to approximately $2\pi \times 0.06$ Å) and scaled to $2 \times 2 \times 1$ for the calculations with double the cell size along $[11\bar{4}]$, as well as on-the-fly generated core-corrected ultrasoft pseudopotentials that treat the $3s^2$ $3p^6$ $3d^2$ $4s^2$ states for Ti and the $2s^2$ $2p^4$ states for O. Calculations using both PBE and where the XC energy is approximated by the local density approximation (LDA)[7] are performed and were found to be consistent. All forces are relaxed to less than 0.01 eV/Å in magnitude using either LBFGS or two point steepest descent (TPSD).

The experimental STEM results allow us to guide the search via constraints: The planes align in both the $[1\bar{1}0]$ and $[11\bar{4}]$ direction and the structure is also well defined up to about 1ML at the center of the GB. We thus perform a constrained search of the interface with a randomization region periodic along the $[1\bar{1}0]$ and $[11\bar{4}]$ directions and of size $2.2 - 2.6$ Å

along the [110] direction. The lattice constants parallel to the grain boundary are chosen to be 13.546 Å and 6.564 Å, based on the lattice constants of rutile TiO_2 found here to be a = 4.641 Å and c = 2.963 Å. The AIRSS approach allows us to search with variable number of atoms to assess different possible stoichiometries at the interface and a large range of stoichiometries are considered (excess Ti atoms given by $2\Gamma_{\mathrm{Ti}} = N_{\mathrm{Ti}} - N_{\mathrm{O}}\frac{N_{\mathrm{Ti}}^{\mathrm{bulk}}}{N_{\mathrm{O}}^{\mathrm{bulk}}} = 0, \pm1, \pm2, +3$, where N_i are the number of atoms in the supercell and bulk structures and the factor of 2 appears because periodic boundary conditions).

The cell is constrained in the $[1\bar{1}0]$ and $[11\bar{4}]$ directions, parallel to the grain boundary, but allowed to relax perpendicular to the grain boundary along [110] to account for grain boundary expansion or contraction. We have tested our approach on the previously studied $\Sigma3\,(112)\,[1\bar{1}0]$ grain boundary in TiO_2[8] and successfully recover the low energy interface structures using our approach.

The results of our search for the $\Sigma13\,(221)\,[1\bar{1}0]$ GB initially give us structures A-E (solid lines in Fig.2 in the main text). Comparison to the experimental images via image simulation yields approximate correspondence of structure B and to some extent structures C and D. These are off-stoichiometric structures with additional Ti atoms at the GB ($\Gamma = 1, 2, 3$). The Ti signal one layer away from the center of the grain boundary at GB = ±1 is however significantly weaker in the experimental images, which leads us to perform further calculations with a system of twice the size along $[11\bar{4}]$ and one Ti atom removed at the GB = ±1 sites. This results in structure E*, an O-rich structure, higher in energy than the lowest energy $\Gamma = -1$ structure we find from our AIRSS calculations. Further inspection shows an O-O dimer at the center of the grain boundary formed due to the removal of Ti atoms at GB= ±1; removing one O atom from each of the O dimers relaxes to structure A*, a stoichiometric structure, significantly lower in energy over a large range of chemical potential than all other structures.

The electronic properties of the grain boundary models were also calculated using a hybrid functional approach implemented in CP2K[9–12] which gives a much more accurate

description of the band gap of TiO_2. These calculations were performed using the Gaussian and plane-waves approach, the PBE0-TC-LRC functional and the auxiliary density matrix approach to enable inexpensive Hartree-Fock exchange.[13,14] The primary basis sets used were of triple-ζ quality with valence and polarisation exponents.[15] The fraction of exact exchange was constrained using the generalised Koopmans condition as described in ref. 16. The approach has been shown to accurately describe the electronic structure of different phases of TiO_2, including a more appropriate description of the band gap differences of rutile and anatase TiO_2 in comparison to calculations based on local density approximation (LDA) or gradient-corrected exchange-correlation functionals such as PBE. This thus allows us to make more direct comparison to experiments and the expected behavior when incorporated in a device. We first consider the band gap of crystalline rutile and anatase TiO_2 shown as solid black and red lines in Fig.5 in the main text and see that, as expected from experiments, the band gap of rutile TiO_2 is approximately 0.2 eV smaller than that of anatase TiO_2.

Ab Initio Thermodynamics

Following the notation from ref. 17, the energetic ordering/the stability of our candidate structures found from searching is assessed by considering the interfacial excess free energy, σ, given for varying stoichiometry as,[4,17]

$$\sigma = \frac{1}{2A}\left(G - N_{\mathrm{Ti}}\mu_{\mathrm{Ti}} - N_{\mathrm{O}}\mu_{\mathrm{O}}\right), \qquad (1)$$

where G is the Gibbs free energy, N_i the number of atoms of species i in the supercell and A the area of the grain boundary, with the factor of 2 appearing because two periodic grain boundaries are in one supercell due to periodic boundary conditions. The chemical potential μ_i is in the range $\mu_i = g_i^0 + (1-\lambda)\,\Delta G^0_{\mathrm{f,\ TiO_2}}$, where $0 \leq \lambda \leq 1$, and $\Delta G^0_{\mathrm{f,\ TiO_2}}$ is the free energy of formation of TiO_2 and g_i^0 the free energy of the bulk structures of i. For bulk Ti we reference to hcp Ti whilst for O we check for both molecular O_2 and solid P63/mmc

oxygen structure. Following ref,[17] the stoichiometry of each structure is expressed in terms of the excess of species,

$$\Gamma_{\mathrm{i}} = \frac{1}{2}\left(N_{\mathrm{i}} - N_{\mathrm{A}}\frac{N_{\mathrm{i}}^{\mathrm{bulk}}}{N_{\mathrm{A}}^{\mathrm{bulk}}}\right), \quad (2)$$

where we here choose to take $i = \mathrm{Ti}$ as the reference species, allowing us to express σ as a function of the chemical potential μ_{Ti}

Figure 7 shows the interfacial excess energy when the local density approximation (LDA) XC functional is employed - this is shifted as expected but consistent with the results shown in the main text in Fig. 2 when the PBE XC functional is used. Similarly the results for the bond-orientational order parameter calculated based on using the LDA XC functional (Figure 8) are consistent with the results shown in the main text in Fig. 4 when the PBE XC functional is used.

EELS calculations

We perform EELS calculations using CASTEP in combination with OptaDOS[18,19] using adaptive broadening.[20] The $L_{2,3}$ Ti edge is considered here since it was found to allow differentiating rutile and anatase behaviour.[21,22] A larger supercell than that used for searching was considered to simulate the bulk between the two grain boundaries more accurately. The original supercell based on doubling the cell size from searching included 336 atoms in the cell; adding an extra 4 layers of bulk between each grain boundary brings the number of atoms to 432 atoms. Given this significant size, we have chosen a plane wave cutoff of 550 eV for computational reasons; we find this to not affect the structural and energetic properties significantly. Otherwise the same parameters as for the refined calculations as described above are employed. A spectral k-point density of 2π 0.04 Å was used. We include a core-hole in our calculations by considering a Ti atom to have an excitation and then average over each plane. To more appropriately compare to experiments we consider here that although we employ a sub-angstrom resolution electron probe we expect a delocalization

factor of approximately 2 Å full width half maximum (FWHM) as well as delocalization due to the specimen thickness of ± 1 atomic plane. We simulate this by broadening our calculated signal assuming a Gaussian function of FWHM = 2 Å perpendicular to the grain boundary plane.

Directly calculating the EELS spectrum is possible but problematic: Proper treatment would require considering spin-orbit coupling at the least, but ideally also treatment of many-electron effects and multiplet effects. This is not trivial and not implemented in many codes (including CASTEP+OptaDos) - and depending on which level of theory is required is very expensive and unfeasible for the large structure we consider here. Instead we calculate the spectrum using non-relativistic, one-electron calculations which then ignores many-electron and multiplet effects as well as spin-orbit coupling. This means only peaks a, b and b' will be reproduced and the relative intensities are only qualitatively reproduced. However, despite this approximation the trend is the same as seen in the experimental results, that is we find bulk rutile and anatase to show the characteristic behavior of the set of peaks b and b', that is a change-over in magnitude. This is shown in Fig. 9 for bulk rutile and anatase and shows that the first sets of peaks are reproduced (the second set is from spin orbit coupling and therefore not included given the level of theory we employ here). The intensities are not correctly reproduced but the two peaks, labelled b and b', are present and shift strength qualitatively similarly as in experiments.

Bond-orientational order parameters

We consider the bond-orientational order parameters as introduced by Steinhardt *et al*[23] - implemented as per Wang *et al.*[24] The Q4, Q6, W4 and W6 parameters are shown as a function of each layer parallel to the grain boundary and we compare this to the known values for bulk rutile and anatase TiO_2 (main text Figure 4). We are primarily interested in the local bonding and therefore consider how Ti atoms bond with the surrounding O atoms by employing a cutoff of 2.34 Å.

Atomic structure of low energy interface structures

Figure 6 shows the bond length analysis and radial distribution function of the theoretical grain boundary structure A*.

Figures 11-19 show the atomic structures of all low energy grain boundary structures - labels as in the plot of the interface energy of the main manuscript Fig.2.

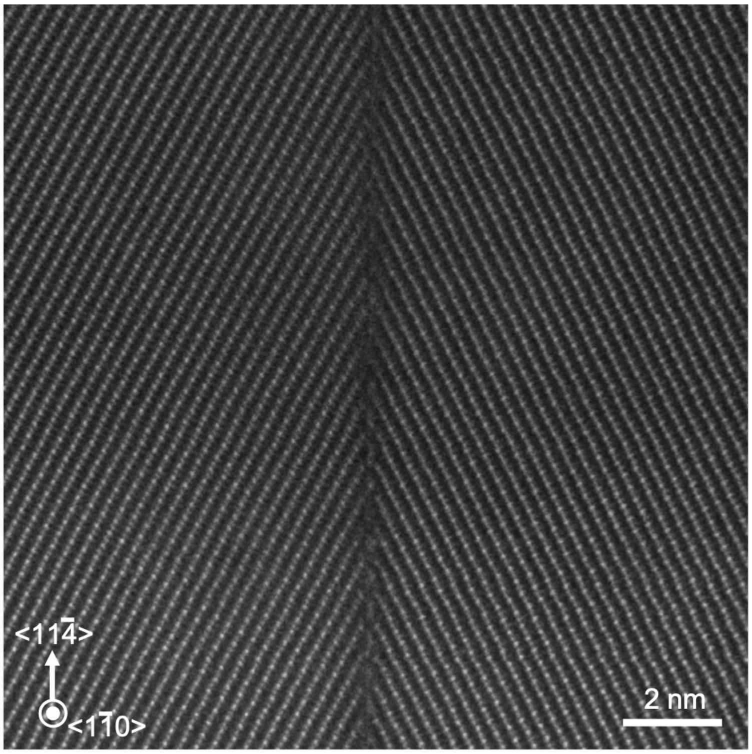

Figure 1: Low-magnification HAADF-STEM images show perfect joining of the bicrystal over a long range.

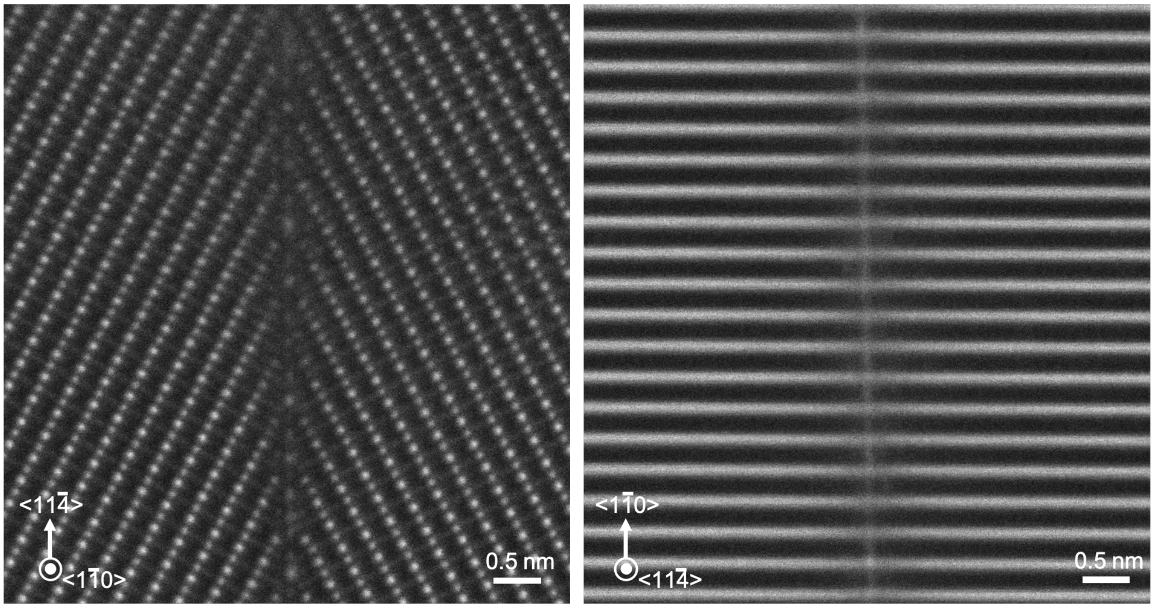

Figure 2: HAADF-STEM images of the grain boundary structure.

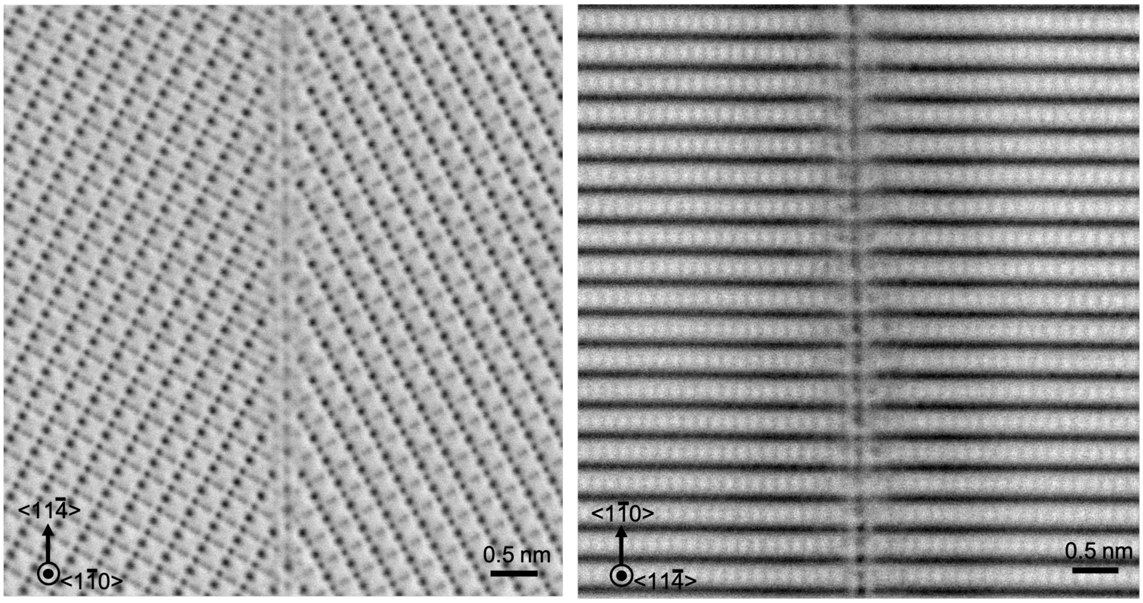

Figure 3: ABF-STEM images of the grain boundary structure.

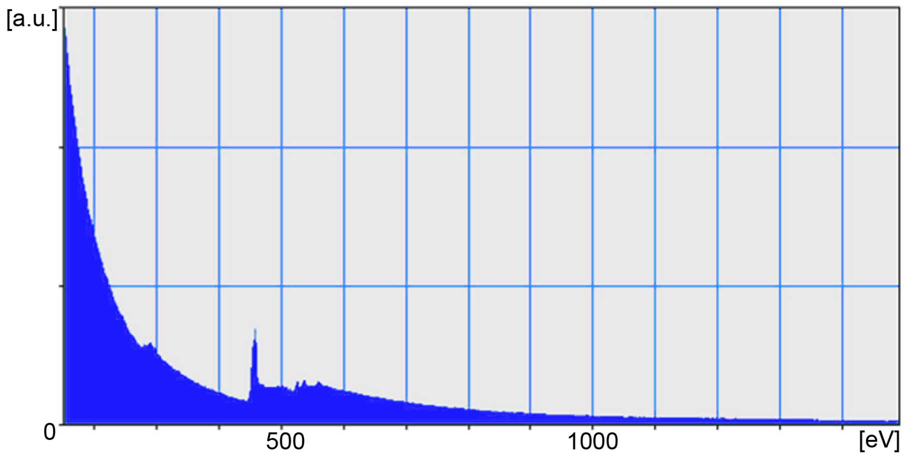

Figure 4: EEL spectra obtained from Σ13 (221) grain boundary. A broad energy range contains the Ti $L_{2,3}$ and O K edges but no substantial peaks from impurities are found.

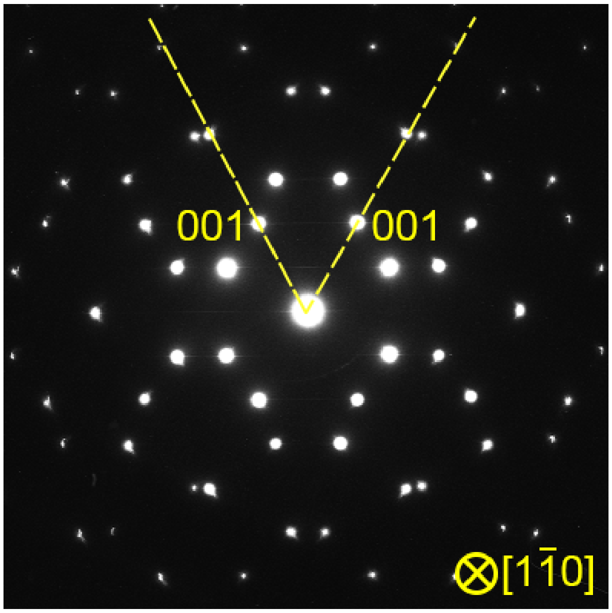

Figure 5: The selected-area diffraction pattern obtained from the Σ13 (221) [1$\bar{1}$0] grain boundary viewed along the [1$\bar{1}$0] direction. Dotted lines indicate the [001] orientation of the upper and lower crystal with an angle of ∼ 57.5°.

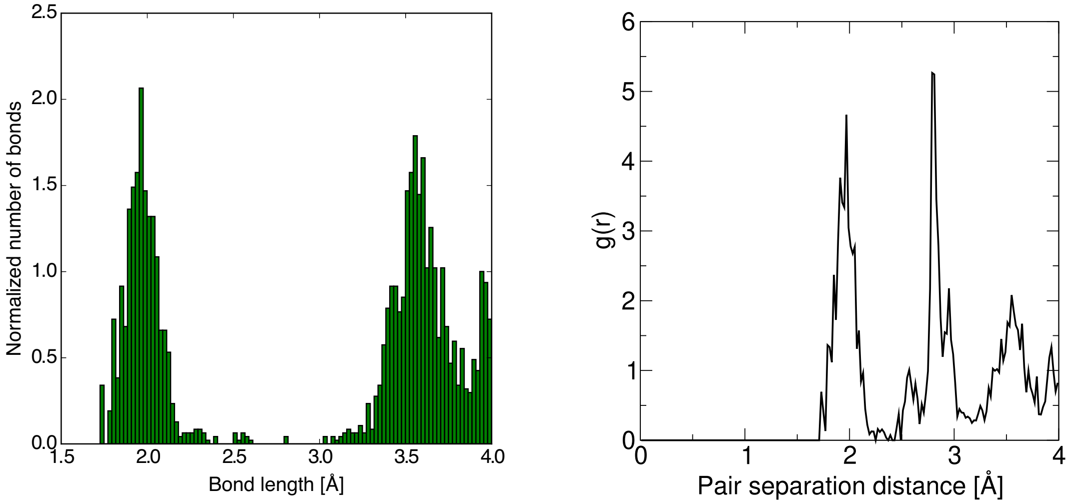

Figure 6: Bond length analysis (Ti bond length to neighboring O atoms) and radial distribution function g(r) of the A* grain boundary structure.

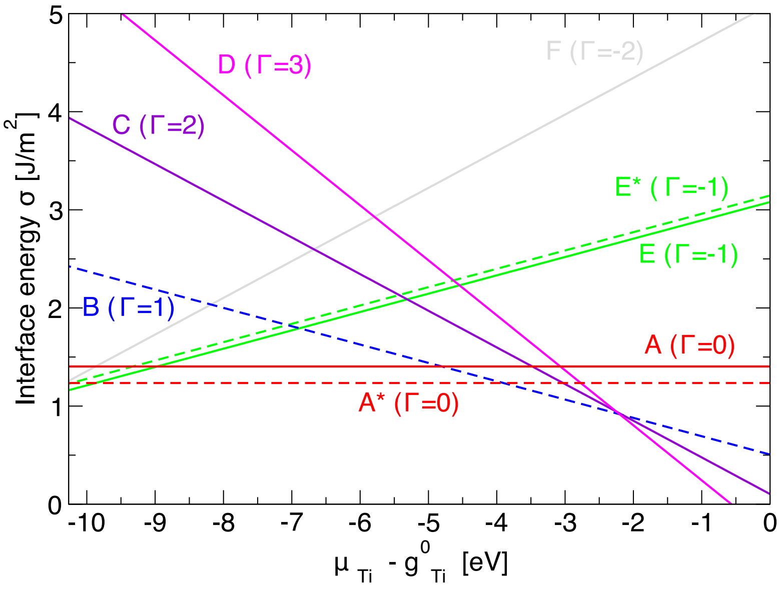

Figure 7: Interfacial energy for the different theoretical structures based on using the LDA XC functional - the results are consistent with the results found using the PBE XC functional.

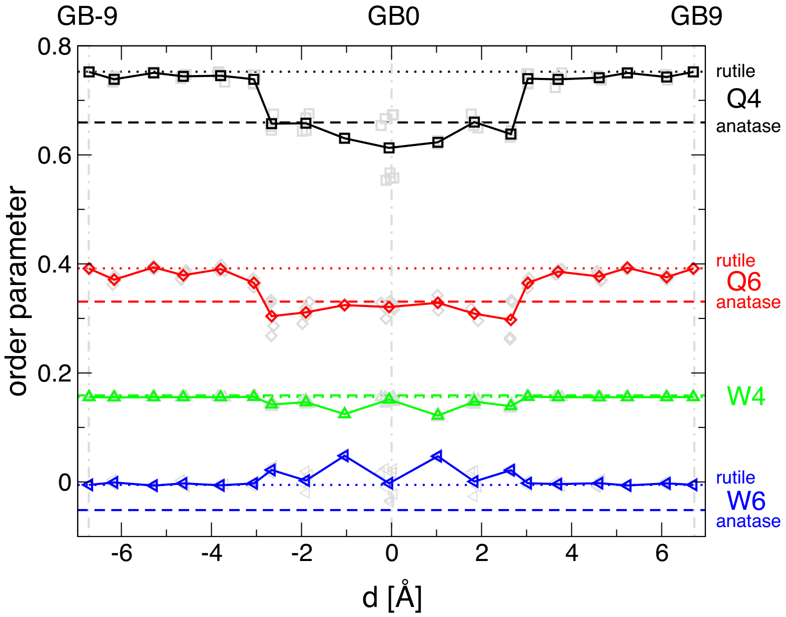

Figure 8: Bond-orientiational order parameter for all Ti atoms of the GB structure based on structure optimized using the LDA XC functional - the results are consistent with the results using the PBE XC functional. Color-coding and further explanation see caption of Fig.4 in the main text.

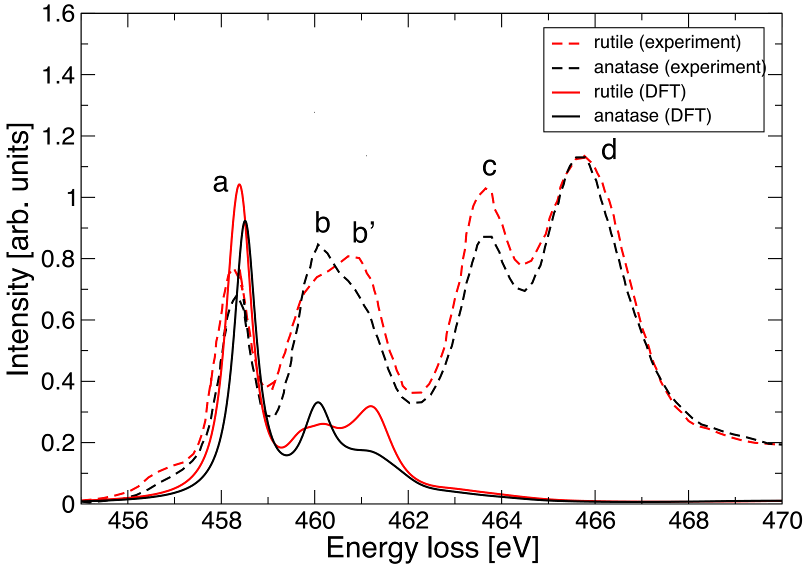

Figure 9: Calculated EELS signal for bulk rutile and anatase TiO_2 using the settings as described in the main text and comparison to experiments (experimental EELS profile extracted from Ref.[21]). Calculated EELS profile is shifted in energy to approximately line up with the experimental results.

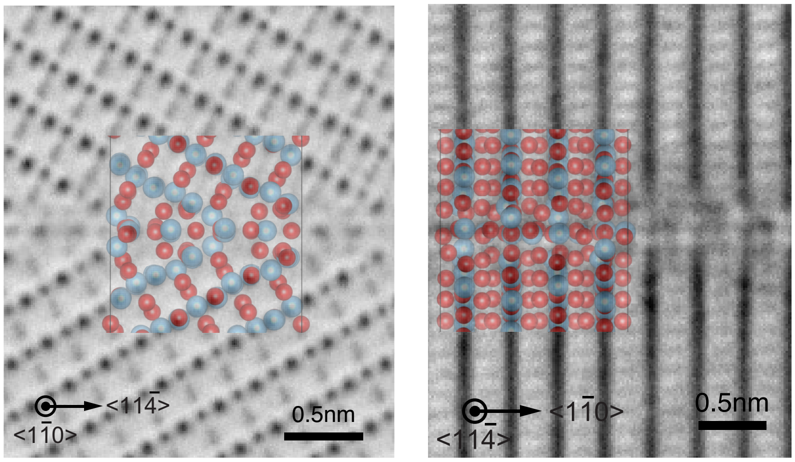

Figure 10: Predicted atomic structure (A*) with lowered opacity on top of ABF images. Orientation as in main manuscript.

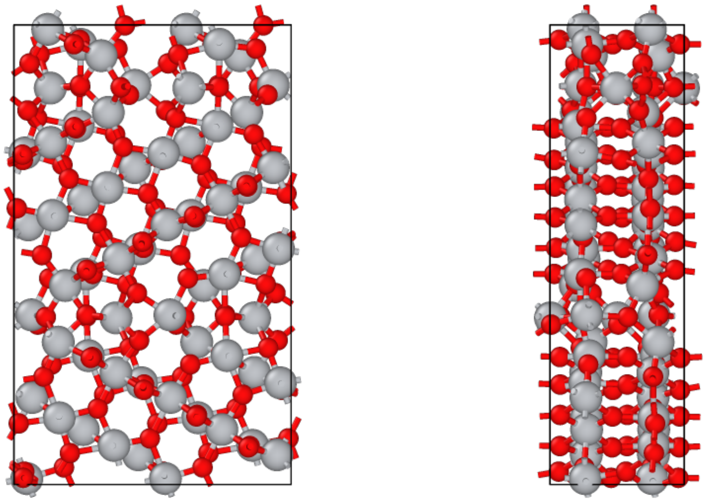

Figure 11: Atomic structure for grain boundary structure A. Directions same as labeled in main manuscript.

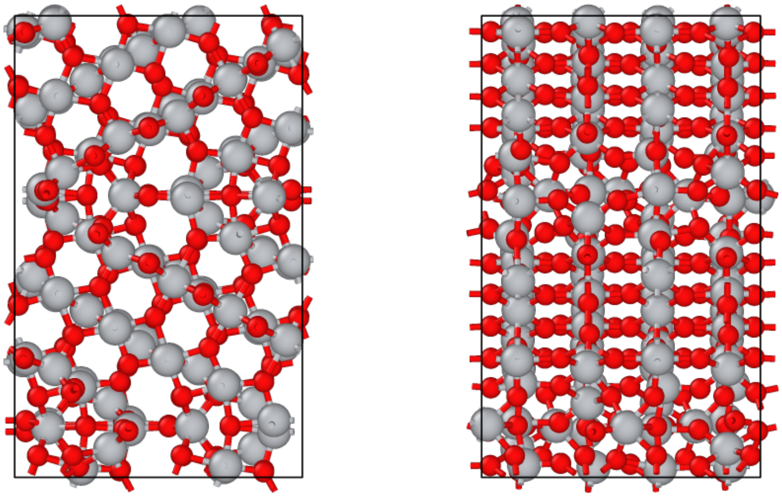

Figure 12: Atomic structure for grain boundary structure A*. Directions same as labeled in main manuscript.

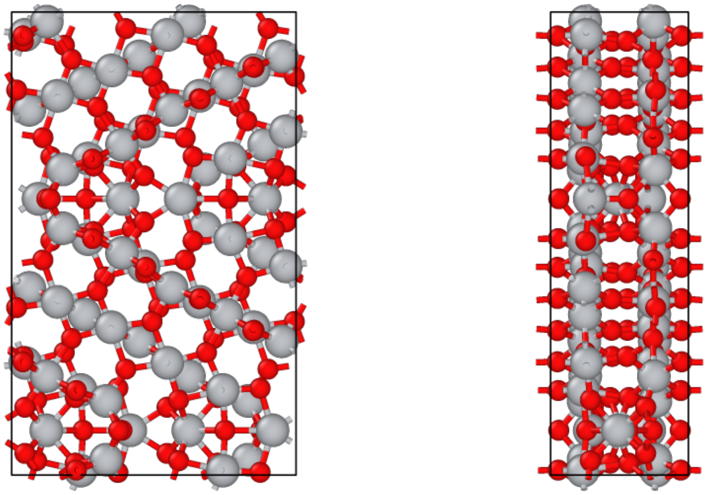

Figure 13: Atomic structure for grain boundary structure B. Directions same as labeled in main manuscript.

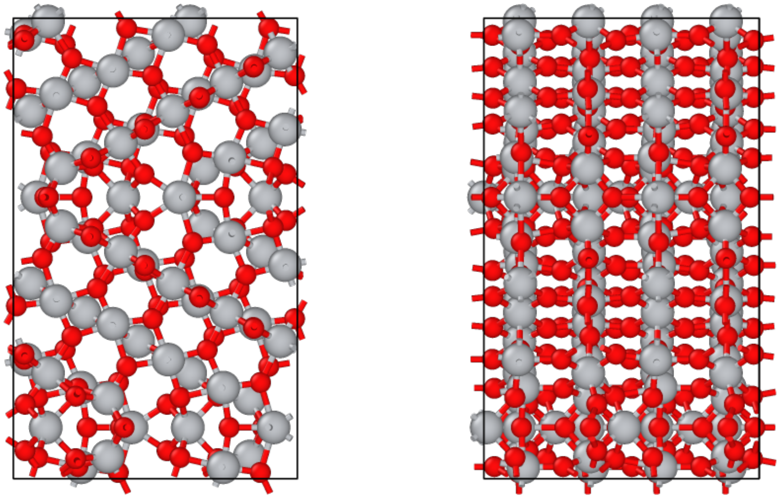

Figure 14: Atomic structure for grain boundary structure B*. Directions same as labeled in main manuscript.

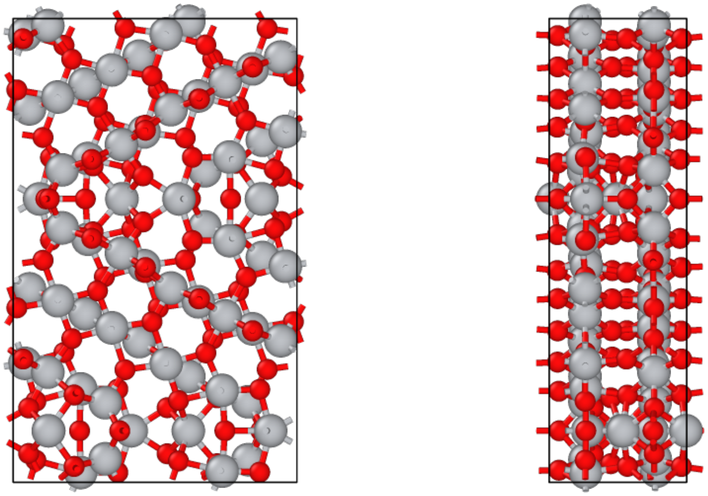

Figure 15: Atomic structure for grain boundary structure C. Directions same as labeled in main manuscript.

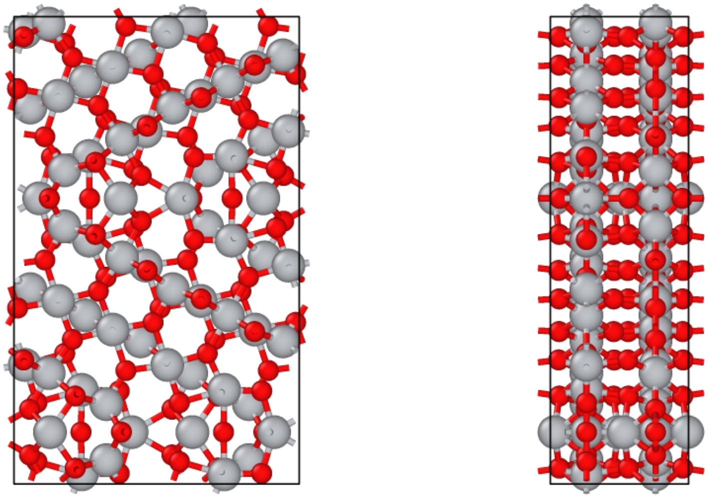

Figure 16: Atomic structure for grain boundary structure D. Directions same as labeled in main manuscript.

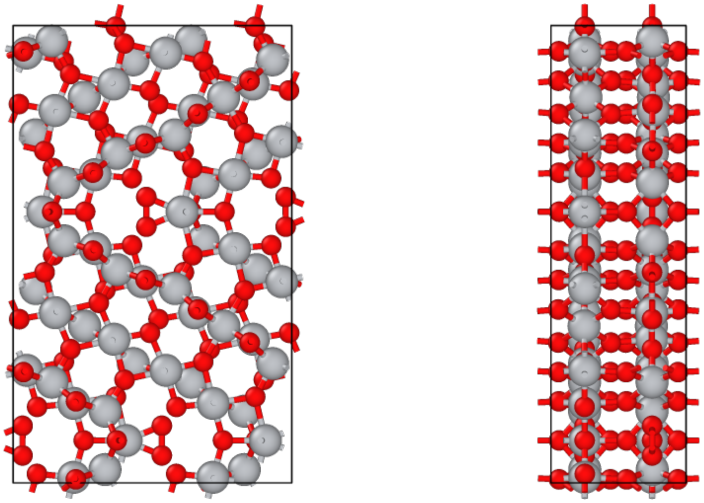

Figure 17: Atomic structure for grain boundary structure E. Directions same as labeled in main manuscript.

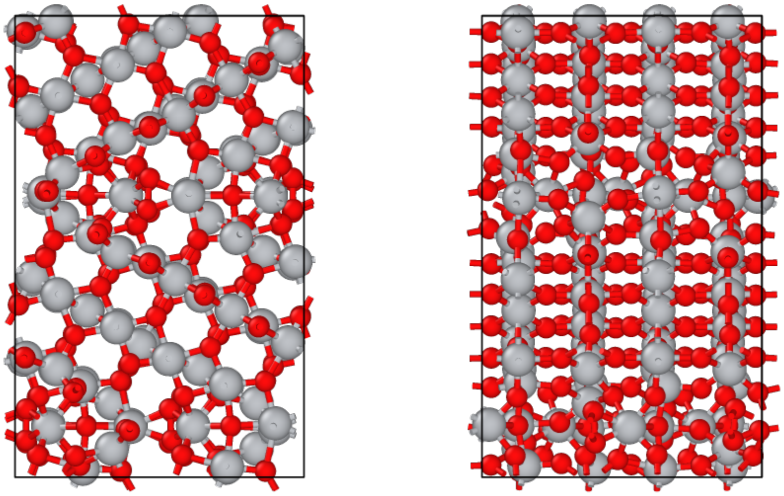

Figure 18: Atomic structure for grain boundary structure E*. Directions same as labeled in main manuscript.

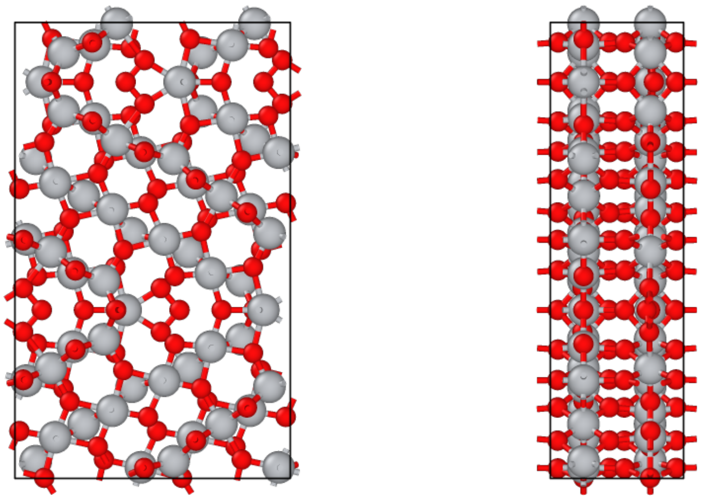

Figure 19: Atomic structure for grain boundary structure F. Directions same as labeled in main manuscript.

References

(1) Kirkland, E. J. *Advanced computing in electron microscopy*; Springer Science & Business Media, 2010.

(2) Pickard, C. J.; Needs, R. J. High-pressure phases of silane. *Physical Review Letters* **2006**, *97*, 1–4.

(3) Pickard, C. J.; Needs, R. J. Ab initio random structure searching. *Journal of physics. Condensed matter : an Institute of Physics journal* **2011**, *23*, 053201.

(4) Schusteritsch, G.; Pickard, C. J. Predicting interface structures: From SrTiO3 to graphene. *Physical Review B - Condensed Matter and Materials Physics* **2014**, *90*, 1–7.

(5) Clark, S. J.; Segall, M. D.; Pickard, C. J.; Hasnip, P. J.; Probert, M. I. J.; Refson, K.; Payne, M. C. First principles methods using CASTEP. *Zeitschrift fur Kristallographie* **2005**, *220*, 567–570.

(6) Perdew, J. P.; Burke, K.; Ernzerhof, M. Generalized gradient approximation made simple. *Phys. Rev. Lett.* **1996**, *77*, 3865–3868.

(7) Perdew, J. P.; Zunger, A. No Title. *Physical Review B* **1981**, *23*, 5048.

(8) Sun, R.; Wang, Z.; Saito, M.; Shibata, N.; Ikuhara, Y. Atomistic mechanisms of nonstoichiometry-induced twin boundary structural transformation in titanium dioxide. *Nature communications* **2015**, *6*, 7120.

(9) VandeVondele, J.; Krack, M.; Mohamed, F.; Parrinello, M.; Chassaing, T.; Hutter, J. Quickstep: Fast and accurate density functional calculations using a mixed Gaussian and plane waves approach. *Comput. Phys. Commun.* **2005**, *167*, 103–128.

(10) Hutter, J.; Iannuzzi, M.; Schiffmann, F.; VandeVondele, J. cp2k: atomistic simulations of condensed matter systems. *Wiley Interdiscip. Rev. Comput. Mol. Sci.* **2014**, *4*, 15–25.

(11) Guidon, M.; Schiffmann, F.; Hutter, J.; VandeVondele, J. Ab initio molecular dynamics using hybrid density functionals. *J. Chem. Phys.* **2008**, *128*, 214104.

(12) Krack, M. Pseudopotentials for H to Kr optimized for gradient-corrected exchange-correlation functionals. *Theor. Chem. Acc.* **2005**, *114*, 145–152.

(13) Guidon, M.; Hutter, J.; VandeVondele, J. Robust periodic Hartree-Fock exchange for large-scale simulations using Gaussian basis sets. *J. Chem. Theory Comput.* **2009**, *5*, 3010–3021, PMID: 26609981.

(14) Guidon, M.; Hutter, J.; VandeVondele, J. Auxiliary density matrix methods for Hartree-Fock exchange calculations. *J. Chem. Theory Comput.* **2010**, *6*, 2348–2364, PMID: 26613491.

(15) VandeVondele, J.; Hutter, J. Gaussian basis sets for accurate calculations on molecular systems in gas and condensed phases. *The Journal of Chemical Physics* **2007**, *127*, 114105.

(16) Elmaslmane, A. R.; Watkins, M. B.; McKenna, K. P. First-Principles Modeling of Polaron Formation in TiO2 Polymorphs. *Journal of Chemical Theory and Computation* **2018**, *14*, 3740–3751, PMID: 29874462.

(17) Chua, A. L.; Benedek, N. A.; Chen, L.; Finnis, M. W.; Sutton, A. P. A genetic algorithm for predicting the structures of interfaces in multicomponent systems. *Nature Materials* **2010**, *9*, 418–422.

(18) Nicholls, R. J.; Morris, a. J.; Pickard, C. J.; Yates, J. R. OptaDOS - a new tool for EELS calculations. *Journal of Physics: Conference Series* **2012**, *371*, 012062.

(19) Morris, A. J.; Nicholls, R. J.; Pickard, C. J.; Yates, J. R. OptaDOS: A tool for obtaining density of states, core-level and optical spectra from electronic structure codes. *Computer Physics Communications* **2014**, *185*, 1477–1485.

(20) Yates, J. R.; Wang, X.; Vanderbilt, D.; Souza, I. Spectral and Fermi surface properties from Wannier interpolation. *PHYSICAL REVIEW B* **2007**, *75*, 195121.

(21) Stoyanov, E.; Langenhorst, F.; Steinle-Neumann, G. The effect of valence state and site geometry on Ti L3,2 and O K electron energy-loss spectra of TixOy phases. *American Mineralogist* **2007**, *92*, 577–586.

(22) Gloter, A.; Ewels, C.; Umek, P.; Arcon, D.; Colliex, C. Electronic structure of titania-based nanotubes investigated by EELS spectroscopy. *Physical Review B - Condensed Matter and Materials Physics* **2009**, *80*, 1–6.

(23) Steinhardt, P. J.; Nelson, D. R.; Ronchetti, M. Bond-orientational order in liquids and glasses. *Physical Review B* **1983**, *28*, 784–805.

(24) Wang, Y.; Teitel, S.; Dellago, C. Melting of icosahedral gold nanoclusters from molecular dynamics simulations. *Journal of Chemical Physics* **2005**, *122*, 1–16.
